# Equatorial auroral records reveal dynamics of the paleo-West Pacific geomagnetic anomaly

**DOI:** 10.1073/pnas.2026080118

**Published:** 2021-05-10

**Authors:** Fei He, Yong Wei, Stefano Maffei, Philip W. Livermore, Christopher J. Davies, Jon Mound, Kaihua Xu, Shuhui Cai, Rixiang Zhu

**Affiliations:** ^a^Key Laboratory of Earth and Planetary Physics, Institute of Geology and Geophysics, Chinese Academy of Sciences, 100029 Beijing, China;; ^b^College of Earth and Planetary Sciences, University of Chinese Academy of Sciences, 100049 Beijing, China;; ^c^School of Earth and Environment, University of Leeds, LS2 9JT Leeds, United Kingdom;; ^d^State Key Laboratory of Lithospheric Evolution, Institute of Geology and Geophysics, Chinese Academy of Sciences, 100029 Beijing, China

**Keywords:** geomagnetic anomaly, ancient aurora, core dynamics, geomagnetic secular variation

## Abstract

Localized regions of low geomagnetic intensity such as the South Atlantic Anomaly allow energetic particles from the Van Allen radiation belt to precipitate into the atmosphere and have been linked to a signature in the form of red aurora–like airglow visible to the naked eye. Smoothed global geomagnetic models predict a low-intensity West Pacific Anomaly (WPA) during the sixteenth to nineteenth centuries characterized by a simple time dependence. Here, we link the WPA to an independent database of equatorial aurorae recorded in Seoul, South Korea. These records show a complex fluctuating behavior in auroral frequency, whose overall trend from 1500 to 1800 AD is consistent with the locally weak geomagnetic field of the WPA, with a minimum at 1650 AD. We propose that the fluctuations in auroral frequency are caused by corresponding and hitherto unknown fluctuations in the regional magnetic intensity with peaks at 1590 and 1720 AD, a time dependence that has been masked by the smoothing inherent in regularized global geomagnetic models. A physical core flow model demonstrates that such behavior requires localized time-dependent upwelling flows in the Earth’s core, possibly driven by regional lower-mantle anomalies.

## Significance

The structure of Earth’s ancient magnetic field is constrained by paleomagnetic measurements. However, owing to data sparsity, models need to be smoothed; such smoothing can obscure rapid but crucial fluctuations in the field. We have shown that historical observations of equatorial aurorae can provide added constraints on the dynamics of Earth’s deep interior, suggesting possible fluctuating behavior of a low-intensity geomagnetic anomaly during the seventeenth and eighteenth centuries in the West Pacific. This anomaly is an ancient analogue to the present-day South Atlantic Anomaly, whose existence impacts the functioning of low-Earth orbit satellites. Our study sheds light on the origin, time dependence, and possible future evolution of similar geomagnetic features.

Variations of Earth’s internally generated magnetic field link motions of the liquid iron core 2,800 km below the planet’s surface to dynamics in near-Earth space. One important feature of the current geomagnetic field is the South Atlantic Anomaly (SAA), a region of low-intensity field strength, which causes energetic particles from the Van Allen radiation belt to penetrate deeper into the atmosphere, damaging low-Earth orbit satellites (1). Recent growth and weakening of the SAA have been linked to changes in the dipole moment (2), while the apparent recurrence of low field intensity in the region has been attributed to the mantle’s influence on core dynamics through the anomalous thermal and chemical structure of the African large low-velocity province (LLVP) (3). Recent advances in model reconstructions (4) have highlighted details of the SAA’s evolution, although scientific understanding, particularly of the processes that govern its dynamics, would be enhanced by better knowledge of other such low-intensity patches. Because the SAA is the only low-intensity patch in the present day, there is therefore a need to seek other patches in archeomagnetic and other related historical records.

**Fig. 1.**
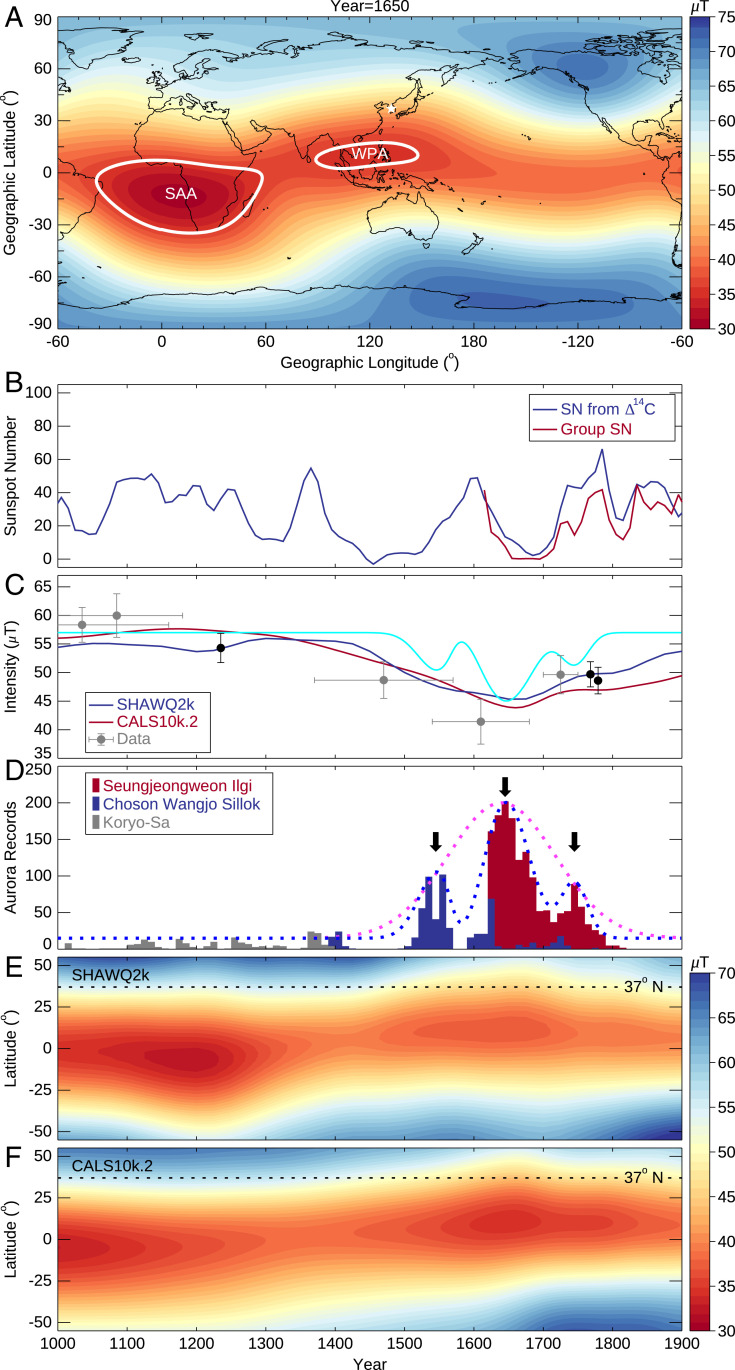
Evolution of magnetic field and Korean auroral records during the period from 1000 to 1900 AD. (*A*) The gufm1 model prediction of the surface magnetic field intensity in 1650 AD, with the SAA and WPA noted by the white contours. The ancient Korean auroral observatory is marked by the white star at 37° N and 127° E. (*B*) Decadal group *SN* (red) and *SN* reconstructed from Δ^14^C data (blue). (*C*) Magnetic intensity at 127° E and 37° N predicted by different geomagnetic field models. The gray and black dots represent archeomagnetic data in South Korea (36° to 38° N and 126° to 128° E) (18) and in Japan (30° to 32° N and 129° to 131° E) (19), respectively. The cyan curve denotes the predicted magnetic field intensity evolution from auroral records (see *Materials and Methods* for details of magnetic field reconstruction). (*D*) Number of auroral records binned in 10 y intervals. The dashed magenta curve denotes a Gaussian function fit of an envelope of the auroral records. The dashed blue curve denotes a multipeak Gaussian function fit of an envelope of the auroral records. The three arrows denote the three peaks. (*E*) Evolution of the magnetic intensity at 127° E predicted by the SHAWQ2k model. (*F*) Evolution of the magnetic intensity at 127° E predicted by CALS10k.2 model.

A notable low-intensity anomaly was centered in the western Pacific Ocean during the seventeenth to eighteenth centuries (Fig. 1*A*), as first shown by the gufm1 model using ship log data (5, 6) (see *SI Appendix*, Fig. S1 showing data coverage), a feature which is also shown in the SHAWQ2k (4) reconstruction based upon an independent global archeomagnetic and volcanic dataset (see *SI Appendix*, Fig. S1 showing data coverage). The West Pacific Anomaly (WPA) is therefore a robust feature which, because global models (4–8) are spatially smoothed, would not be present in the reconstructions if not required to fit to the data. The unavoidable temporal smoothing used in all models suggests a 300 y period of low intensity, although the evolution of the WPA on shorter timescales cannot be ruled out and is a focus of investigation in this study. Geographically, the WPA is located close to the western edge of a region of anomalous lower-mantle structure: the LLVP in the Pacific. Interestingly, the SAA is also on the western edge of a similar anomaly, the African LLVP, which has been conjectured to promote upwelling structures (3). This suggests, like the SAA, that the WPA could also be a mantle-driven feature.

Because the WPA has received much less attention than the SAA in the literature, here, we briefly describe the emergence and basic characteristics of the WPA. Our description is based on the CALS10k.2 model (7), which is equivalent to the gufm1 model for 1600 AD onwards, although we note that this is an inevitably smoothed version of the true evolution due to many factors, including data sparsity and the need for global regularization, and also, the fact that the deep-sea and lacustrine sediment data from which it is built innately time averages the recorded field on much longer timescales than examined in this study. In 1400 AD, a low-intensity anomaly stretched from the Atlantic Ocean eastward to the Indonesian Archipelago, with its minima located west of the Gulf of Guinea (*SI Appendix*, Fig. S2). The WPA separated from the main anomaly between 1540 and 1550 AD and its core, located above the South China Sea between the Philippine Archipelago and current-day Vietnam, reached its minimal intensity around 1650 AD (Fig. 1 *E* and *F*). After this epoch, the clockwise rotation of the south polar high-intensity patch caused the WPA to shrink until disappearance after 1800 AD and, concurrently, the main Atlantic low-intensity anomaly moved further west.

## Results

### Equatorial Aurora in the WPA.

The structure of the internally generated magnetic field has profound consequences for Earth’s atmosphere. Close to low-intensity regions of magnetic field, precipitation of energetic particles can reach ∼100 km altitude (9, 10) and produces a unique type of dynamic red equatorial aurora in the night sky, which is totally different from the traditional chemical-related stable red airglow in regions of more typical magnetic intensity. As the result of particle deposition during the times of geomagnetic disturbance, the red equatorial aurora exhibits polar aurora–like dynamic signatures and is significantly stronger than the traditional red airglow (10).

Northward of the WPA, ancient dynasties in China, Korea, and Japan preserved numerous official/unofficial astronomical records, of particular importance here being the official daily records in ancient Korea around the seventeenth century. The most frequently recorded night sky glow was described as “vapors like fire light,” which has been widely interpreted as red auroras (11–15). However, previous ancient auroral catalogs and investigations mainly focused on the red auroras that occurred northward of the Korean peninsula (i.e., the equatorward expansion of the northern lights). Such records account for less than one-third of the auroral records in Korean historical books. Recently, through systematic searches within three Korean historical books, a new catalog containing 2,013 auroral records from 1012 to 1811 AD was published (16), enabling us to constrain the evolution of the WPA (see *SI Appendix*, Fig. S3 and *Materials and Methods* for the description of the ancient Korean auroral records in detail).

Among the 2,013 auroral records, 1,422 were observed southward of the Korean peninsula. The equatorward expansion of the northern auroral oval does not account for the large amount of red aurora southward of the Korean peninsula since the northern auroral oval can rarely expand to such low latitudes in East Asia even under extreme space weather conditions (17). Traditional red nightglow in East Asia is in most cases invisible to the unaided eye on the ground even under the conditions of high solar activity and geomagnetic storms (see *Materials and* *Methods* for the analysis of visibility of traditional red airglow). However, in the vicinity of the WPA, the weak magnetic field strength promoted enhanced energetic particle precipitation during geomagnetic storms, likely strengthening the emission intensity of the equatorial aurora to a level that it became visible to unaided eye on the ground (see *SI Appendix*, Figs. S4–S8 and *Materials and Methods* for the analyses of the visibilities of the traditional red airglow and red equatorial auroral in ancient Korea). Solar forcing was likely not great enough to expand the polar aurora to Korean latitudes but strong enough to induce equatorial auroral emissions in the areas with low magnetic field intensities. We found that observations of the equatorial aurora were frequently noted to the south of the Korean peninsula, close to the WPA, suggesting that the temporal and spatial variations of the equatorial aurora might be used as a proxy of the evolution of the WPA as explained below.

Fig. 1 *B*–*F* present the temporal evolution of the solar activity, the auroral records, and the predicted magnetic field intensity from 1000 to 1900 AD; the archeomagnetic intensity data are extracted from refs. 18 and 19. Special attention is paid to the period between 1500 and 1800 AD, when 1,785 out of the 2,013 auroral events were recorded. Among the 1,785 records, 1,327 (74%) were observed to the south of the Korean peninsula, indicating that the vast majority of the auroral records between 1500 to 1800 AD in Fig. 1*D* are equatorial aurora. The first key observation is that the maximum of the envelope (Fig. 1*D*, magenta dotted curve) of the auroral sightings at 1650 AD corresponds with a reduced field intensity at Seoul (Fig. 1*C*), shown not only by coeval minima in both CALS10k.2 and SHAWQ2k models but also by the trend in the available regional data. The localized weakening of the field at Seoul is caused by the northward expansion of the WPA (Fig. 1 *E* and *F*). However, the equatorial auroral records show a much more complex time dependence than the simple trend of the global geomagnetic field models, exhibiting fluctuations with three peaks at 1545, 1645, and 1745 AD (see the blue dashed curve and the three arrows in Fig. 1*D*) and punctuated by low values at 1590 and 1720 AD.

The enhanced particle precipitation that causes the aurorae can stem from either (or both) increased solar activity or a locally weakened magnetic field. Fig. 1*B* shows that the auroral peaks are decoupled from the decadal group sunspot number (*SN*) (20) and *SN* reconstructed from Δ^14^C data (21), which suggests that an origin in secular variation (SV) of solar activity is unlikely. Instead, we hypothesize that the maxima and minima of the auroral signal are caused by, respectively, minima and maxima in the intensity of the regional magnetic field, which do not appear in global geomagnetic models based on data currently available because of data sparsity and smoothing. Thus, we propose that both the magnetic intensity at Seoul (where the aurorae were observed) and at the nearby WPA have maxima at 1590 and 1720 AD, and minima at 1545, 1645, and 1745 AD. However, because of spatial gradients in the field, the intensities at Seoul and the WPA are assumed to be offset. Our proposed intensity evolutions for Seoul, South Korea, and the WPA (see *Materials and Methods* for the details of magnetic field reconstruction) are shown as the cyan curve in Fig. 1*C* and the curves in *SI Appendix*, Fig. S9, respectively.

**Fig. 2.**
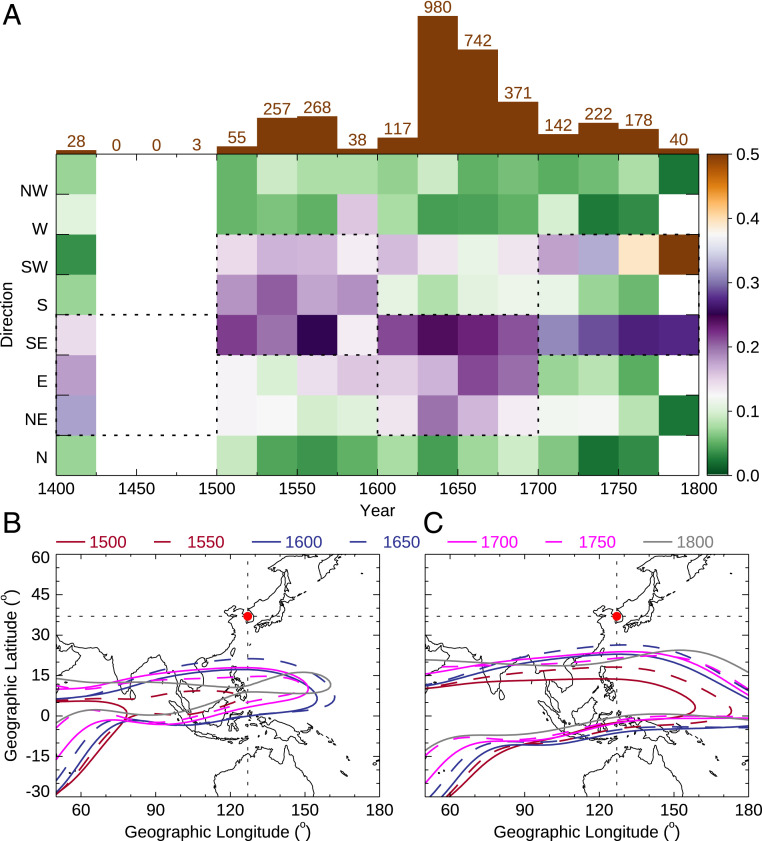
Evolution of the directions of the Korean red aurora and the WPA. (*A*) Histogram of auroral events (*Top*) and the probability of occurrence of the Korean red aurora sighted in different directions in 25 y bins (*Bottom*). (*B*) CALS10k.2 model predictions of the areas of the WPA with intensity lower than 36,000 nT. (*C*) CALS10k.2 model predictions of the areas of the WPA with intensity lower than 38,000 nT. Contours in different colors and line styles represent different years with an increment of 50 y from 1500 AD. The red dot represents the observation location of the red equatorial aurora.

The directions of the equatorial auroral records between 1500 and 1800 AD also exhibit clear centennial evolution (Fig. 2*A* and *Materials and Methods*, which presents a calculation of the probability of occurrence of different directions). The directions concentrated around the south during the sixteenth century, shifted to the southeast and east during the seventeenth century, and finally turned to the southwest and southeast during the eighteenth century, as highlighted by the dashed rectangles in Fig. 2*A*. Such a variation is generally consistent with the eastward and northward expansion of the WPA as predicted by the CALS10k.2 model (Fig. 2 *B* and *C*) (see also *SI Appendix*, Fig. S2 for maps of magnetic field intensity every 100 y). According to the model predictions, the low-intensity magnetic anomaly began to expand northward and eastward after 1400 AD, and the model predicted the largest area of the anomaly, and presumably the minimum magnetic intensity, around 1650 AD (Fig. 2 *B* and *C*), when the number of auroral records achieved a maximum. After 1650 AD, the WPA began to shrink and split with the magnetic field intensity southeast of the Korean peninsula keeping at a relatively low level (see gray line in Fig. 2 *B* and *C*) and resulting in a relatively large probability of occurrence of the red equatorial aurora at southeast direction.

### Core Dynamics Explanation of the WPA.

We now consider whether the proposed evolution of the WPA (*SI Appendix*, Fig. S9), as suggested by the equatorial auroral records, has a plausible core dynamics explanation. According to our proposed intensity variation of the WPA, the fastest rates of intensity change *Ḟ* have an amplitude of about 100 nT ⋅ y^−1^ and occur at around 1570 and 1610 AD. This is comparable to (but at the upper end of) *Ḟ* from a range of historical geomagnetic field models [see *SI Appendix*, Table S5 of Korte and Constable (22)]. Similar but smaller *Ḟ* would be required to explain the fluctuations in the WPA model at 1680 and 1760 AD.

**Fig. 3.**
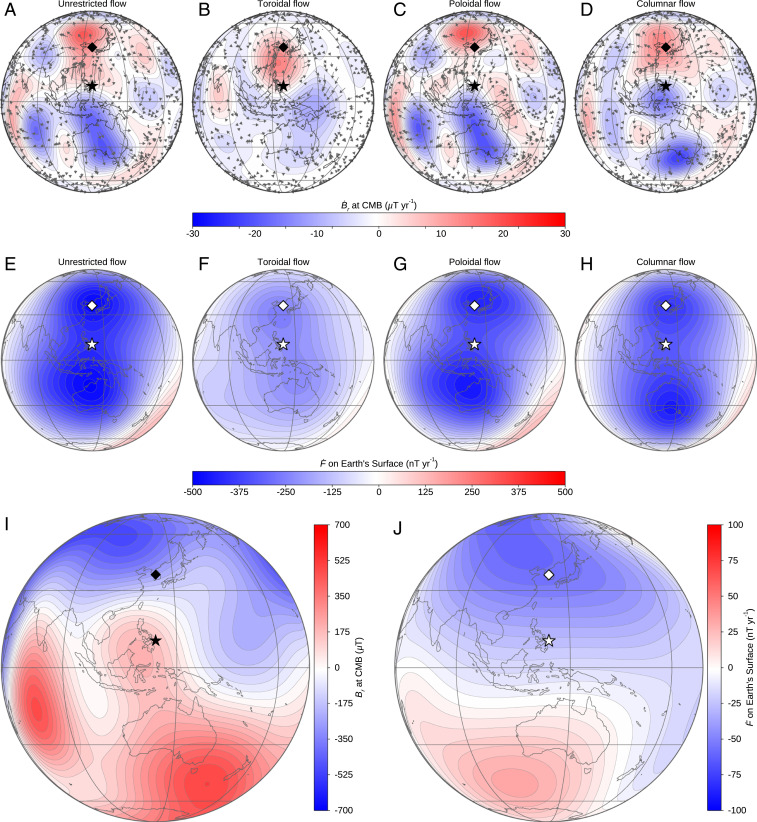
Optimized flow geometries and their SVs. (*A*–*D*) The flow structure on the CMB (arrows) overlaid by contours of the radial SV *Ḃ*_*r*_. (*E*–*H*) The corresponding rate of intensity change *Ḟ* on Earth’s surface. These optimal solutions are calculated from the radial magnetic field at the CMB (*I*) and *Ḟ* on Earth’s surface (*J*), according to the CALS10k.2 model, in 1590 AD. On the Earth’s surface, the center point of the WPA and the location of the auroral observations in Korea are marked as white stars and diamonds in *E*–*H* and *J*; projections of these locations onto the CMB are shown by black stars and diamonds in *A*–*D* and *I*.

We now examine whether the maximum values in |*Ḟ*| of 100 nT ⋅ y^−1^ were physically possible, by calculating the fastest possible variations in *F* that could have been driven by flows at the surface of the core acting upon the global structure of the magnetic field. Rather than investigate the possible dynamics at the two separate epochs of 1570 and 1610 AD, we focus attention on the field at their average age, 1590 AD, since the large-scale structure of the field given by global models changes only slightly over the 40 y period. We apply the optimization algorithm presented in Livermore et al. (23), with the radial magnetic field at the core–mantle boundary (CMB) given by CALS10k.2 (Fig. 3*I*) and the SHAWQ2k (*SI Appendix*, Fig. S10) models, assuming a rms flow speed of 13 km ⋅ y^−1^, also used in Livermore et al. (23) and in line with modern core flow inversions (see *Materials and Methods* for the mathematical details of the optimization algorithm). These flows are optimal in the sense that they supply the most negative *Ḟ* (i.e., the largest possible |*Ḟ*|) at the center point of the WPA (127° E and 10° N). In this analysis, we neglect magnetic diffusion on the grounds that it is likely to be subdominant to the effects of core flow on decadal–centennial timescales; calculations also show that horizontal magnetic diffusion has a negligible effect (see *Materials and Methods* for the details of the optimal flow calculations). Motivated by different possible dynamical regimes in the core (see ref. 23), we impose one of the following geometrical constraints: 1) unrestricted flow, 2) purely toroidal flow, 3) purely poloidal flow, and 4) columnar flow. For each of the cases (1 to 4), any optimized |*Ḟ*| less than the target value of 100 nT ⋅ y^−1^ would rule out the particular geometry. The purely toroidal flow is motivated by the proposed stratified layer at the top of the core allowing only horizontal motions; the purely poloidal flow allows upwellings consistent with flux expulsion; and the columnar flow is imposed by demanding equatorial symmetry and is motivated by the likely structure of flows causing decadal dynamics within the core (24). Table 1 shows the optimized *Ḟ* for each type of core flow model, based on the global field at 1590 AD according to either CALS10k.2 or SHAWQ2k. We evaluate each to spherical harmonic degree 10 and ignore any effects due to unresolved smaller-scale fields. All the values of *Ḟ* are negative, consistent with a deepening of the WPA and therefore with an increase in aurorae. Importantly, in terms of absolute value, all the optimized |*Ḟ*| are above 100 nT ⋅ y^−1^, showing that any of the core flow models can instantaneously create the required intensity change. The largest |*Ḟ*| correspond to the unrestricted flows and the lowest to the purely toroidal flows which, for the assumed field geometry, clearly are not as effective in generating SV. As the lower rows of Table 1 show, |*Ḟ*| according to the CALS10k.2 and SHAWQ2k models are much smaller than those inferred from the auroral records, consistent with the smoothed evolution of the WPA as shown in Fig. 1*C*.

**Table 1.** Optimized *Ḟ* at the center point of the WPA

Core flows	*Ḟ* (nT ⋅ y^−1^)*	
	SHAWQ2K	CALS10k.2
Unrestricted flow	−396	−397
Toroidal flow	−207	−156
Poloidal flow	−338	−365
Columnar flow	−315	−322
Reduced toroidal	−103.5	−78.0
Model	−4.0	−23.3
Auroral record	∼−100	

* Assuming core flows with the given form (left column) and based on global field models at 1590 AD according to SHAWQ2k and CALS10k.2 to spherical harmonic degree 10. The “Reduced toroidal” row contains results of a calculation with a toroidal flow constrained to be one-fourth of the kinetic energy. The rates of change derived from the smoothed global models themselves, along with the rate of change inferred from the auroral records, are given in the last rows.

Fig. 3 *A*–*H* shows the optimized flows and the corresponding *Ḃ*_*r*_ at the CMB and *Ḟ* on the Earth’s surface. The images for the unrestricted, poloidal, and columnar cases are similar (Fig. 3 *A*, *C*, and *D*), showing horizonal divergence (upwelling) located underneath Indonesia and South Korea, whereas the purely toroidal flow shows a two-vortex structure converging under the Philippines (Fig. 3*B*). The images of *Ḃ*_*r*_, which show the effect of the flow on the magnetic field in all cases, have a positive (red) patch under Korea and a negative (blue) patch to the south of the optimization location (the white stars in Fig. 3), consistent in sign with the local structures derived from smoothed geomagnetic field models (*SI Appendix*, Fig. S2). Because these regions occur where *Ḃ*_*r*_ has opposite sign to *B*_*r*_ itself (Fig. 3 *I* and *J*), the overall effect is to reduce the localized radial field on the CMB and therefore also deepen the WPA on the Earth’s surface.

The optimized *Ḟ* at the WPA are high upper bounds, in that they assume that all of the kinetic energy of the surface flow is available to cause the localized change (whereas, in practice, only a modest fraction would be available to drive localized flows). Indeed, the optimal flows displayed in Fig. 3 have a footprint of at most one-fourth of the total CMB area. If we assume that the total kinetic energy is uniformly distributed across the surface of the core, we then need to restrict the available kinetic energy for the localized flow to one-fourth of the global kinetic energy, and we obtain (see “Reduced toroidal” row in Table 1) optimal *Ḟ* for the purely toroidal flows to be, at most, ∼100 nT ⋅ y^−1^ for CALS10k.2 and SHAWQ2K. Because the flow is unlikely to attain this optimal structure, this suggests that the purely toroidal flow cannot explain the fluctuating signal inferred from the auroral records. As illustrated in Fig. 3, optimal flows that are not purely toroidal, all include horizontally diverging structures, suggesting that upwelling flows may be key to explaining the proposed fluctuations: Indeed, one explanation of the auroral records is a succession of core flow upwellings, one every hundred years or so, each followed by a downwelling or other mechanism to restrengthen the field. Such dynamics stand in stark contrast with core flow inversions for present-day SV which suggest low flow magnitudes under the Pacific with only weak upwellings, including in the area under Korea (25). Numerical geodynamo (26–29) and thermal convection (30, 31) simulations show that upwelling (downwelling) structures can be driven by locally low (high) heat flux that renders the local core fluid hot (cold), dynamics ultimately driven by laterally varying thermal structure in the lower mantle. Paleo- and archeomagnetic datasets over the last 3,000 y suggest that patches of reversed radial magnetic field preferentially emerge close to the LLVPs located beneath the Pacific and Africa (32), a behavior that can be explained by expulsion of a toroidal magnetic field, consistent with broad upwelling regions beneath the LLVPs. Given the current presence of reverse flux patches beneath the southern Atlantic Ocean, it seems reasonable that similar structures and low-intensity anomalies were present in eastern Asia in the past, as our results suggest. In fact, the magnetic field intensity predicted by the CALS10k.2 model shows recurrent low-intensity anomalies in Southeast Asia.

## Discussion

The equatorial auroral observations in ancient Korea and core flow simulations are consistent with the existence and evolution of an ancient WPA, as predicted by the smoothed global geomagnetic field models. The cause of the auroral signal we link to the WPA is ultimately driven by enhanced particle precipitation. Although we exclude correlations with sunspots and large solar storms, we cannot rule out variations in minor yet still enhanced solar forcing within the 300 y period. In this paper, we assume a constant rate of enhanced solar forcing and attribute the fluctuations of the auroral signal to the corresponding fluctuations in the geomagnetic field strength of the WPA, with peaks of intensity at 1590 and 1720 AD. Such a centennial timescale is consistent with the statistical features of reversed flux patches during the last millennium (33); thus, like the SAA, the WPA likely originated from similarly driven reversed flux patches. More archeomagnetic data prior to 1800 AD in the South China Sea, the region of Southeast Asia, and the Bay of Bengal are essential for characterizing the evolution of the WPA. The changes in occurring directions in aurorae for each 100 y are also consistent with the features of reversed flux patches suggested by previous studies (34). The centennial timescale of the WPA is much shorter than the 700 y recurrence feature of the more long-lived SAA (3). We conjecture that the WPA originates as an expulsion of reversed flux from a smaller eddy than those responsible for the SAA at the top of the core (giving centennial behavior and shorter lifetime). This smaller eddy may be suggestive of different local effects within the outer core beneath the Pacific compared to the Atlantic driven by regional stratification or different structures in the lowermost mantle.

## Materials and Methods

### Ancient Korean Auroral Records.

The red auroral catalog was constructed from three Korean official historical books: *Koryo-Sa* (918 to 1391 AD, *gao li shi* in Chinese or *History of Koryo*), *Choson Wangjo Sillok* (1392 to 1910 AD, *chao xian wang chao shi lu* in Chinese or *The Veritable Records of the Choson Dynasty*), and *Seungjeongweon Ilgi* (1623 to 1910 AD, *cheng zheng yuan ri ji* in Chinese or *The Daily Records of the Royal Secretariat of Joseon Dynasty*). All these books are written in Chinese. The auroral phenomena were recorded in the books as “*qi ru huo*” or “*qi ru huo guang*” (vapors like fire or vapors like fire light), “*chi qi*” (red vapors), “*ru huo qi*” (fire-like vapors), and “*chi jin*” (odd red vapors). A total of 2,013 auroral records during the period from 1012 to 1811 AD were cataloged in the dataset (16). All the aurorae were observed at Seoul, South Korea. An example of the auroral record is shown in *SI Appendix*, Fig. S3. The scanned copy of this record can be publicly accessed at the National Institute of Korean History (http://sjw.history.go.kr/id/SJW-A24020230-00200). English translations of the key information are shown in the red squares, and the corresponding pronunciation of the Chinese are shown in the square brackets in *SI Appendix*, Fig. S3*A*. The date, local time (LT), and direction of the aurora was specifically recorded, and their definitions are shown in *SI Appendix*, Fig. S3 *B* and *C*. Before 1400 AD, only 203 records were compiled, and most of the recorded LT and direction information were imperfect. Therefore, the 1,810 auroral records after 1400 AD are considered in this study. It is noted in Fig. 1*D* that the auroral records are punctuated by low values at 1590 and 1720 AD. The dataset is complete, except for the loss of documents in the *Choson Wangjo Sillok* during 1570 to 1590 AD, and therefore, the three peaks with gaps in between are a reflection of auroral signal rather than an artifact of data collection.

The red airglow emission occurs at altitudes mostly between 150 and 350 km. For an observer at geographic latitude of θ (θ=37° at Seoul) on the ground, the maximum distance between the observer, S, and the point in the sky from which the aurora is most visible, P, is along the line of sight (LOS) tangential to the Earth’s surface (*SI Appendix*, Fig. S3*D*) and is calculated as follows. We consider a spherical model of the Earth with a mean radius of *R*_E_ = 6371 km as shown in the cut view of *SI Appendix*, Fig. S3*D*. First, we calculate the distance along the LOS for an aurora event located in the local southward direction. For simplicity, we can therefore consider the meridian plane passing through the observer, S. For Seoul, it is the meridian plane at geographic longitude of φ=127°. The length of the line segment SP in *SI Appendix*, Fig. S3*D* is the greatest, and the corresponding integrated intensity is the strongest at *α* = 90°, where *α* is the viewing angle between the local vertical in S and the LOS direction. In this case, and the maximum latitudinal difference λ satisfies (*R*_E_+*h*)cosλ=*R*_E_, let *R*_E_+*h = R*_P_; the coordinates of the point P are given by *R*_P_(cos(θ-λ)cosφ, cos(θ-λ)sinφ, sin(θ-λ)). Then, the line segment SP is rotated around the local zenith (the direction OS in *SI Appendix*, Fig. S3*D*) by 360° to get the LOS distance at different directions, as shown by the blue curve for *h* = 350 km in *SI Appendix*, Fig. S3*E*. For other altitudes, the maximum LOS distance can be calculated in the same manner (e.g., the LOS distance for altitude of 150 km is shown by the cyan curve in *SI Appendix*, Fig. S3*E*).

### Visibility of Traditional Red Airglow.

Here, we show that the traditional red airglow mechanisms are not responsible for Korean auroral observations. The term “traditional airglow” refers to emissions that are purely excited through chemical/photochemical reactions in the atmosphere and ionosphere. Evaluating the unaided human eye response to nightglow is complex. The visual sensation is mainly determined by two factors: the spectral response function of the eye and the spectral intensity of the nightglow event.

According to the International Commission on Illumination standard illumination function for photopic vision (*SI Appendix*, Fig. S4), the human eye is most sensitive to photons at 555 nm for photopic vision. In the circumstance of auroral emission at nighttime, a dark-adapted eye would apply this photopic vision. Rose (35) obtained a visual sensation with 100 photons ⋅ s^−1^. Using an 8 mm pupil for the eye and 2 arcmin for a pixel, it can be calculated that the photon flux required to stimulate a physical sensation is 200 Rayleigh (36). Under the same illumination condition, only a brightness greater than 200 Rayleigh can be perceived by the eye at other wavelengths. For example, the thresholds are 750 Rayleigh at 630 nm, 2,000 Rayleigh at ∼650 nm [OH(6 → 1) Meinel band], 50,000 Rayleigh at ∼700 nm [OH(7 → 2) Meinel band], and 800,000 Rayleigh at ∼740 nm [OH(8 → 3) Meinel band].

Given the occurrence of polar auroras in low magnetic latitude areas (such as the Korean peninsula) during some extreme space weather events, the frequent occurrence of redline aurora southward of Korea is surprising. The most likely interpretation is a red equatorial aurora based on the following analysis. For a redline airglow phenomena occurring southward of Korea, we consider nightglow emissions from the oxygen atoms and hydroxyl (OH) as a possible mechanism for the excitation of red auroras.

We first consider the emissions at 630 nm from the oxygen atoms. In absence of energetic particle streams and under a steady-state condition, the dissociative recombination loss of O_2_^+^ gives rise to the traditional redline emission at 630 nm in the ionosphere and thermosphere at night (37). The volume emission rate (VER) *VER*_630_ can be expressed by the following equation (38):

VER630=0.756f(D1)k3[O2][O+]1+(k2[N2]+k5[O2]+k6[e−]+k7[O])/A1D photon cm-3 s-1,k2=2.0×10−11e111.8/Tn cm3 s-1,k3=3.23×10−12e1118/Ti−1.68×105/Ti2 cm3 s-1,k5=2.9×10−11e67.5/Tn cm3 s-1,k6=1.6×10−12Te0.91 cm3 s-1,k7=9.2×10−13 cm3 s-1, [1]

where *k*_2_, *k*_3_, *k*_5_, *k*_6_, and *k*_7_ are reaction rate coefficients, *f*(^1^D)=1.1 is the quantum yield, *A*_1D_ = 7.45 × 10^−3^ ⋅ s^−1^ is the Einstein coefficient, *T*_*i*_ is the ion temperature, *T*_*e*_ is the electron temperature, *T*_*n*_ is the neutral temperature, [e^−^] is the electron density, [O^+^] is the oxygen ion density, [N_2_] is the molecular nitrogen density, [O_2_] is the molecular oxygen density, and [O] is the atomic oxygen density. It is assumed that *T*_*i*_=*T*_*e*_=*T*_*n*_, which is a good approximation at night for low- to midlatitudes. Eq. **1** indicates that the redline airglow is primarily generated in the ionospheric *F* region. The height-dependent plasma and neutral parameters in Eq. **1** are calculated using the International Reference Ionosphere 2016 (IRI2016) (39) and the NRLMSIS-00 empirical atmosphere model (40), respectively. Both models use solar radio flux at 10.7 cm (*F*_10.7_ index in sfu, 1 sfu= 10^−22^ W ⋅ m^−2^ ⋅ Hz^−1^), as a proxy for the solar activity. For the solar activity during the period 1500 to 1800 AD, the *SN* is less than 70. Based on a linear relationship between the *SN* and the *F*_10.7_ index during low activity (41), *F*_10.7_ = 0.26 × *SN* + 68.75, it is inferred that the corresponding *F*_10.7_ index is less than 90 sfu. We designed two categories of simulations to reveal the variations of traditional nightglow emissions during the period 1500 to 1800 AD. In the first category, we set *F*_10.7_ = 90 sfu and Kp = 2, representing a weak space weather condition. As a comparison, a relatively strong space weather condition with *F*_10.7_ = 110 sfu and Kp = 5 is set for the second category. For each category, three representative seasons (equinox, summer solstice, and winter solstice) and three LTs (22 h, 0 h, and 2 h) are considered. All the simulations are carried out in the meridian plane at 127° E and 0° to 40° N with the IRI2016 and NRLMSIS-00 models and the observing geometry defined in the previous section. For a ground observer at 127° E and 37° N, when looking to the south at different viewing angles (*α* = 0° for local nadir and *α* = 90° for horizon, see *SI Appendix*, Fig. S3 for the definitions), the variations of the redline emission intensities are shown in *SI Appendix*, Fig. S5. It is shown that, even under active solar conditions of *F*_10.7_ = 110 sfu and Kp = 5, the maximum intensity at viewing angle of 90° is less than 600 Rayleigh, weaker than the human eye threshold of 750 Rayleigh at 630 nm, except for the case in *SI Appendix*, Fig. S5*F* with LT = 22 h. This implies that generally the traditional redline airglow at 630 nm cannot be observed on the ground during 1500 to 1800 AD.

We now consider an alternative excitation mechanism. The OH emission arises from the rotational–vibrational transitions between different vibrational levels (*v* ≤ 9) of the ground-state X^2^Π between 80 and 95 km (42). In the red portion of the visible spectrum, three Meinel bands need to be considered, namely the 620 to 640 nm OH (6 → 1) band, the 675 to 705 nm OH (7 → 2) band, and the 720 to 750 nm OH (8 → 3) band. In a climatological view, the kinetic parameters for OH nightglow modeling for the *v* ≥ 5 states are adopted (43, 44). Between 80 and 95 km altitudes OH(*v*) is primarily produced from the H + O_3_ reaction through the following kinetic mechanism:

$$\text{O}\;+\;{\text{O}}_{2}\;+\;\text{M}\;\to \;{\text{O}}_{3}\;+\;\text{M};\;\;\;\;\text{M}\;=\;{\text{N}}_{2},\;{\text{O}}_{2},\;\text{O},$$ [2]

$${\text{O}}_{3}\;+\;\text{H}\;\to \;\text{OH}\left(v^{\prime }\leq 9\right){\;+\;\text{O}}_{2},$$ [3]

$$\text{OH}\left(v^{\prime }\right){\;+\;\text{O}}_{2}\;\to \;\text{OH}\left(v^{\prime \prime }\right){\;+\;\text{O}}_{2},$$ [4]

$$\text{OH}\left(v^{\prime }\right){\;+\;\text{N}}_{2}\;\to \;\text{OH}\left(v^{\prime \prime }\right){\;+\;\text{N}}_{2},$$ [5]

$$\text{OH}\left(v^{\prime }\right)\;+\;\text{O}\;\to \;\text{OH}\left(v^{\prime \prime }\right)\;+\;\text{O,}$$ [6]

$$\text{OH}\left(v^{\prime }\right)\;\to \;\text{OH}\left(v^{\prime \prime }\right)\;+\;hv\text{.}$$ [7]

Then, the production rate *P*_OH_ of the vibrationally excited OH for all vibration levels is

$$P_{OH}=k_{1}\left[\text{O}\right]\left[{\text{O}}_{2}\right]\left[{\text{O}}_{2}\right]\;+\;k_{2}\left[\text{O}\right]\left[{\text{O}}_{2}\right]\left[{\text{N}}_{2}\right].$$ [8]

The rate constants are *k*_1_ = 6.2 × 10^−34^ (*T*_*n*_/300)^−2.0^ cm^6^ ⋅ s^−1^ for M = O_2_ and *k*_2_ = 5.7 × 10^−34^ (*T*_*n*_/300)^−2.8^ cm^6^ ⋅ s^−1^ for M = N_2_. The contribution from M = O is very small below 95 km and is neglected. The production rate of vibrational state at *v* is

$$P_{9}=f_{9}P_{OH},$$ [9a]

$$P_{v}\left(v\leq 8\right)=f_{v}P_{OH}+\sum_{j=1}^{9-v}\left[n_{v+j}A_{v+j,v}+n_{v+j}\left(k_{3,\left(v+j,v\right)}\left[{\text{O}}_{2}\right]+k_{4,\left(v+j,v\right)}\left[{\text{N}}_{2}\right]\right)\right],$$ [9b]

where *A*_*v*+*j*,*v*_ is the Einstein coefficient (44), *f*_*v*_ is the branching ratio for the *v*th state in reaction (3), and *k*_3_ and *k*_4_ are state-to-state quenching rate constants (42). Loss of excited OH includes spontaneous emission of radiation as qualified by the Einstein coefficients and quenching by collisions with O_2_ or N_2_. The loss rate is

$$L_{v}=\sum_{j=0}^{v-1}A_{v,j}+\sum_{j=0}^{v-1}\left(k_{3,\left(v,j\right)}\left[{\text{O}}_{2}\right]+k_{4,\left(v,j\right)}\left[{\text{N}}_{2}\right]\right).$$ [10]

Then, assuming chemical equilibrium, the number density of the *v*th state is

$$n_{v}=\frac{P_{v}}{L_{v}}.$$ [11]

Finally, the VER for the *v*′→*v*″ vibrational transition is

$$VER_{v^{\prime }v^{\prime \prime }}=E_{v^{\prime },v^{\prime \prime }}A_{v^{\prime },v^{\prime \prime }}n_{v^{\prime }},$$ [12]

where *E*_*v*′*v*″_ is the photon energy for the *v*′→*v*″ vibrational transition.

Eqs. **8**–**12** form a theoretical model for the OH nightglow emission. The neutral density and temperature profiles are simulated using the NRLMSIS-00 model. We now repeat the intensity calculations for this alternative mechanism. The solar activity, season, and LT conditions are the same as those used to calculate the 630 nm emission. The emission intensities of the OH (6 → 1) band, the OH (7 → 2) band, and the OH (8 → 3) band are shown in *SI Appendix*, Fig. S6, respectively. It is shown that even under the condition of *F*_10.7_ = 110 sfu, the maximum intensities at a viewing angle of 90° are less than 600 Rayleigh for the OH (6 → 1) band, less than 1,000 Rayleigh for the OH (7 → 2) band, and less than 3,000 Rayleigh for the OH (8 → 3) band, far weaker than the human eye thresholds of 2 kg/Rayleigh, 50,000 Rayleigh, and 800,000 Rayleigh, respectively. This implies that generally the traditional OH airglow in the red portion of the visible light cannot be observed on the ground during 1500 to 1800 AD.

### Visibility of Equatorial Aurora in Ancient Korea.

According to the calculations in the above section, the traditional red airglow cannot be seen by unaided human eye on the ground. There should be another energy source to excite the upper atmosphere to generate stronger emissions that can be seen by unaided human eye on the ground. Analogous to the equatorial aurora occurring at the SAA (10), we propose that the energy source is the energetic particle precipitation due to the weak magnetic field strength at the WPA.

Based on limited satellite measurements, it is inferred that the energy flux of the low-energy electron precipitation (*E* < 23.8 keV) is 0.1 to 1 ergs ⋅ cm^−2^ ⋅ s^−1^ at the SAA during relatively geomagnetically quiet periods. During geomagnetic storms, the energy flux will increase at least one order of magnitude relative to the quiet time level, achieving 1 to 10 ergs ⋅ cm^−2^ ⋅ s^−1^ (44). The resultant auroral emission rate is proportional to the influx of energetic electrons. Observations and simulations (45) demonstrated that the peak VER varies from 10 to 200 photon ⋅ cm^−3^ ⋅ s^−1^ at 630 nm for energy flux of 1 to 10 ergs ⋅ cm^−2^ ⋅ s^−1^.

By analogy with the SAA, we assume a Gaussian-type distribution of the total input energy flux versus geographic latitude in the meridian plane at a geographic longitude of 127° E. The latitudinal profile of the field strength predicted by the gufm1 in 1650 AD is shown in *SI Appendix*, Fig. S7*A*, with the minimum at the WPA center (10° N). The corresponding Gaussian-type distributions of total input energy flux peak are at the WPA center (*SI Appendix*, Fig. S7*B*). The total input energy flux is limited below 0.1 ergs ⋅ cm^−2^ ⋅ s^−1^ at latitudes higher than 50° in both hemispheres. Since the redline aurora favors low‐energy electron precipitation, we further assume a Maxwellian-type electron precipitation with characteristic energy of 2 keV and a maximum energy flux of 1, 3, and 10 ergs ⋅ cm^−2^ ⋅ s^−1^ (blue, red, and black, respectively, in *SI Appendix*, Fig. S7*B*). When the energy flux profile is input into the global airglow (GLOW) model (46) in companion with the IRI2016 and NRLMSIS-00 models, we obtain the simulated latitudinal distributions of the VER that are shown in the left panels of *SI Appendix*, Fig. S8. The variations of redline emission intensities versus viewing angles are shown in the right panels of *SI Appendix*, Fig. S8 for a ground observer in Korea (37° N and 127° E).

For the cases of total input energy flux 3 and 10 ergs ⋅ cm^−2^ ⋅ s^−1^, the slant intensity exceeds the human eye threshold of 750 Rayleigh at viewing angles greater than 87° and 67° as shown in *SI Appendix*, Fig. S8 *B* and *D*, respectively. When the peak energy flux is less than 1 ergs ⋅ cm^−2^ ⋅ s^−1^, the case becomes similar to normal cases shown in *SI Appendix*, Fig. S5. This implies that equatorial auroras can be visible to unaided human eye during geomagnetic storms. For lower magnetic field strength (i.e., larger area) in the WPA and stronger geomagnetic storms, the precipitating energy flux becomes stronger and the redline emission intensity will be even stronger accordingly. Thus, the equatorial aurora could have more frequently been observed in ancient Korea.

### Prediction of Magnetic Field Evolution.

Based on the hypothesis that the fluctuations in the auroral records are caused by changes in the local magnetic field intensity rather than solar activity, we present a reconstruction of the regional magnetic variation in the following way. First, we fit a multi-Gaussian function to create a smooth envelope of the auroral records from Seoul in Fig. 1*D*. We then assume that the magnetic field intensity at Seoul, *F*(*t*), and the auroral frequency, *h*(*t*), are empirically related by *F*(*t*) = *A*/(1+*Bh*(*t*)), where *t* is time in years. Implicit in this formulation is that a weakened field causes enhanced particle precipitation. The two constants, *A* and *B*, are determined by 1) a low aurorae value (*h* = 0) with an intensity of 57 μT (1400 AD in Seoul, South Korea, Fig. 1*C*) and 2) the minimum intensity of 45 μT at 1645 AD (consistent with the CALS10k.2 and SHAWQ2k models and the data point centered at 1610 AD), which is coeval with the maximum value of *h*. The predicted intensity evolution is shown in Fig. 1*C* (cyan curve).

For the center of WPA, we assume an intensity evolution of the form $\tilde{F}\left(t\right)=\tilde{A}/\left(1+\tilde{B}h\left(t\right)\right)$, where the values of $\tilde{A}$ and $\tilde{B}$ are determined by a maximum of *h* = 0 at 1400 AD of 39 μT and a minimum intensity of 34 μT at 1645 AD, respectively, corresponding to the maximum value of *h*. The predicted intensity evolution is shown in *SI Appendix*, Fig. S9.

### Probability of Occurrence of Different Directions.

Since most of the auroral records in *Koryo-Sa* have no direction information (203 records out of the 2,013 records before 1400 AD), only the 1,810 records after 1400 AD are plotted in Fig. 2. To increase the significance of the statistics, the daily auroral records are binned into 25 y intervals. For each daily auroral record, the aurora observed at the same direction but at different LTs are counted as one event, and the aurora observed at different directions are counted as individual events in the statistics. An auroral record may contain multiple auroral events. The histogram of the auroral events is shown at the top of Fig. 2*A*. Another requirement is that the total number of auroral events in a 25 y bin is greater than eight to ensure that there is at least one event at each direction on average. Then, for each 25 y bin, the occurrence probability at a specific direction is calculated dividing the number of auroral events by the total number of events in the bin.

### Optimal Flow Calculations.

In order to estimate whether magnetic field intensity variations of 100 nT ⋅ y^−1^ are reasonable for 1590 AD, we calculated the most extreme rate of change allowed by fluid flows on top of the fluid outer core. We did so by calculating optimized fluid flows using the algorithm presented in ref. 24 that we briefly describe here. Ignoring magnetic diffusion, the evolution of the magnetic field at the top of the core is described by the SV equation:

$${\dot{B}}_{r}=\frac{dB_{r}}{dt}=-\nabla_{H}\left(u_{H}B_{r}\right),$$ [13]

where *B*_*r*_ is the radial component of the magnetic field, **u**_*H*_ are horizontal flows close to the surface of the outer core, described in a spherical coordinate system (*r*, θ, and φ), and ∇_*H*_ is the horizontal component of the gradient operator. The magnetic field intensity rate-of-change *Ḟ=dF*/*dt* can be calculated from the SV coefficients of the spherical harmonics expansion of *Ḃ* and *B*. The evolution equation for *Ḟ* then reads as the following:

$$\dot{F}=\frac{dF}{dt}=G^{T}q,$$ [14]

where **q** are the coefficients of the modal expansion of **u**_*H*_,

$$u_{H}=\sum_{k}q_{k}u_{k},$$ [15]

and **G** is a column vector that depends upon *B*. The modes **u**_*k*_ are divergence-free toroidal and poloidal horizontal modes, respectively written as $\nabla \times \left[Y_{l}^{m}\left(\theta ,\varphi \right)r\right]$ and $\nabla_{H}\left[Y_{l}^{m}\left(\theta ,\varphi \right)r\right]$, with $Y_{l}^{m}\left(\theta ,\varphi \right)$ being Schmidt seminormalized spherical harmonics and *k* a shorthand expression for the degree and order (*l* and *m*). The flows shown in Fig. 3 are optimal in the sense that they maximize *Ḟ* under the assumption that the SV coefficients evolve according to Eq. **13** and with a given value *T*_0_ for the rms flow **u**_*H*_. Mathematical manipulations (24) lead to the following formulas for the sought values of **q**:

$$q=\frac{1}{2\lambda }E^{-1}G,$$ [16]

where *E*=*l*(*l*+1)(2*l*+1)^−1^ is a normalized factor, and λ is adjusted so that the rms value of the optimal flow is set to *T*_0_. The optimal **q** and *Ḟ* values are linear functions of *T*_0_, whereas the structure of the optimal flows is independent of it.

In our calculations, we set *T*_0_ = 13 km ⋅ y^−1^ (23). To restrict the flows to toroidal or poloidal only, we simply set to zero the **q** coefficient for the poloidal and toroidal modes, respectively. To restrict the flow to columnar geometry, we constrain the coefficients **q** so that the optimal flow satisfies the equatorial symmetries *u*_*φ*_ = *u*_*φ*_(π-θ) and *u*_*θ*_ = −*u*_*θ*_(π-θ).

In our calculations, the coefficients of the background magnetic field *B* are given by the CALS10k.2 model, truncated at spherical harmonic degree *L*_*OBS*_ = 10. The optimal flow is truncated at order *L*_*U*_ = 25, in order to ensure convergence of the results (23). The components of the optimal SV **Ḃ** are truncated at degree *L*_*SV*_ = 35.

The effect of horizontal magnetic diffusion on the optimal solution has been investigated and, for realistic values of electrical conductivity for the outer core material, found to contribute less than 0.1% of the diffusion-free values, in agreement with previous results (23).

## Supplementary Material

Supplementary File

## Data Availability

The ancient Korean auroral records used in this study are publicly available at Figshare (https://doi.org/10.6084/m9.figshare.14471154). The GLOW model (version 0.982) is publicly available at https://www2.hao.ucar.edu/modeling/glow/code. All other study data are included in the article and/or *SI Appendix*.
