# Supplementary material for: Equatorial auroral records reveal dynamics of the paleo-West Pacific geomagnetic anomaly
Source: Proc Natl Acad Sci U S A. 2021 May 10;118(20):e2026080118. doi: 10.1073/pnas.2026080118 (PMC8157951; doi:10.1073/pnas.2026080118)
Supplement: Supplementary File [file pnas.2026080118.sapp.pdf]

PNAS
www.pnas.org

Supplementary Information for

Equatorial auroral records reveal dynamics of the paleo-West Pacific geomagnetic anomaly

Fei He[a,b], Yong Wei[a,b,1], Stefano Maffei[c,1], Philip W. Livermore[c], Christopher J. Davies[c], Jon Mound[c], Kaihua Xu[a,b], Shuhui Cai[d], Rixiang Zhu[d]

Yong Wei, Stefano Maffei.
Email: weiy@mail.iggcas.ac.cn, S.Maffei@leeds.ac.uk

This PDF file includes:

Figures S1 to S10

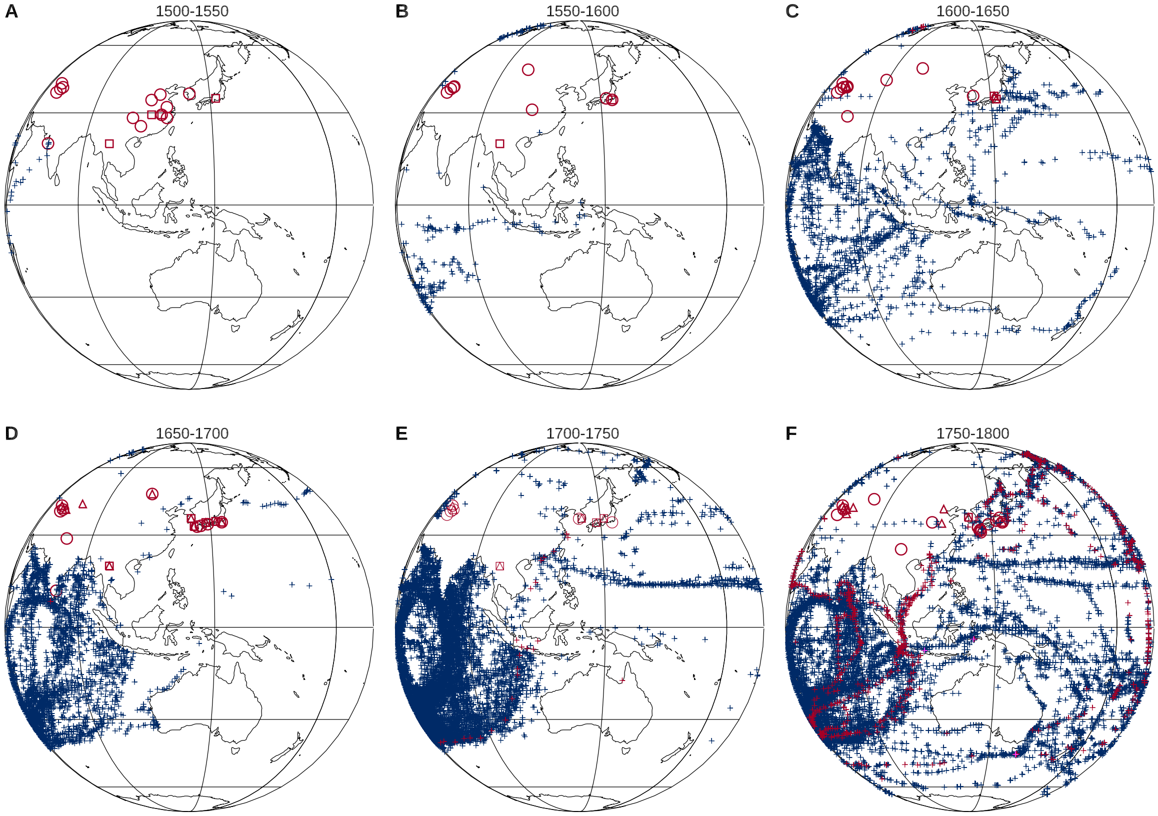

Fig. S1. Ship log and archeomagnetic data between 1500 and 1800 AD. (**A**) 1500 to 1550 AD. (**B**) 1550 to 1600 AD. (**C**) 1600 to 1650 AD. (**D**) 1650 to 1700 AD. (**E**) 1700 to 1750 AD. (**F**) 1750 to 1800 AD. The circles, squares, and triangles represent intensity, declination and inclination data, respectively, from the databases used in CALSK10k.2 and SHAWQ2k. The blue, red, and pink crosses denote the declination, inclination, and intensity data from the ship logs used in the construction of the *gufm*1 model.

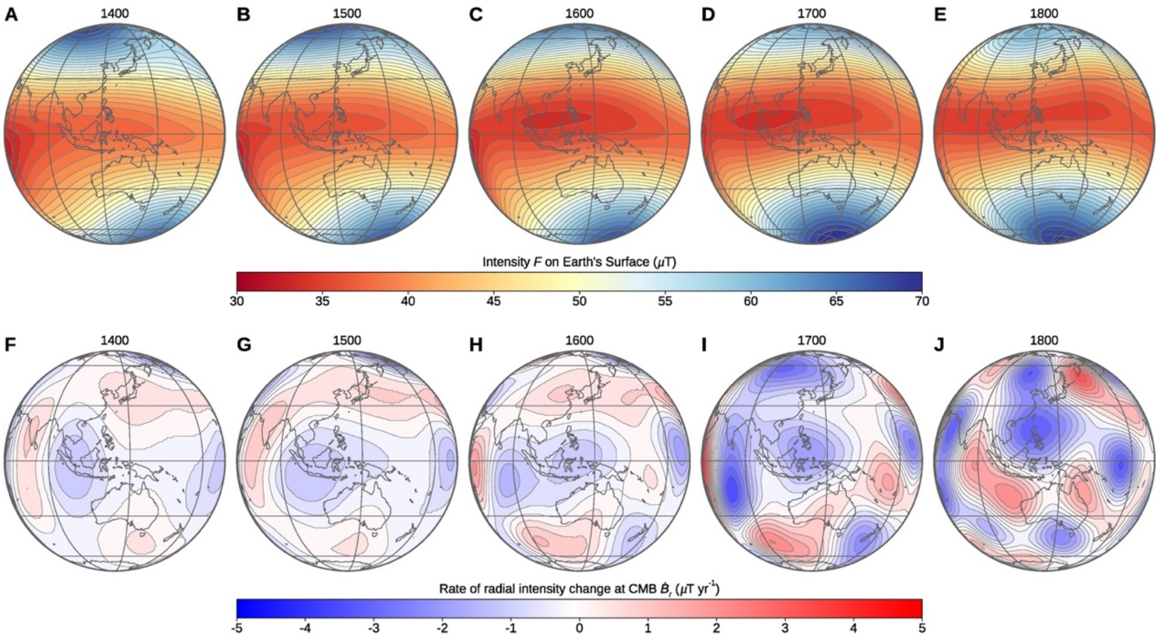

Fig. S2. Evolution of the WPA at different epochs from the CALS10k.2 model. (**A**–**E**) Snapshots of intensity F on Earth's surface. (**F**–**J**) Rate of radial intensity change $\dot{B}_r$ at the core-mantle boundary (CMB).

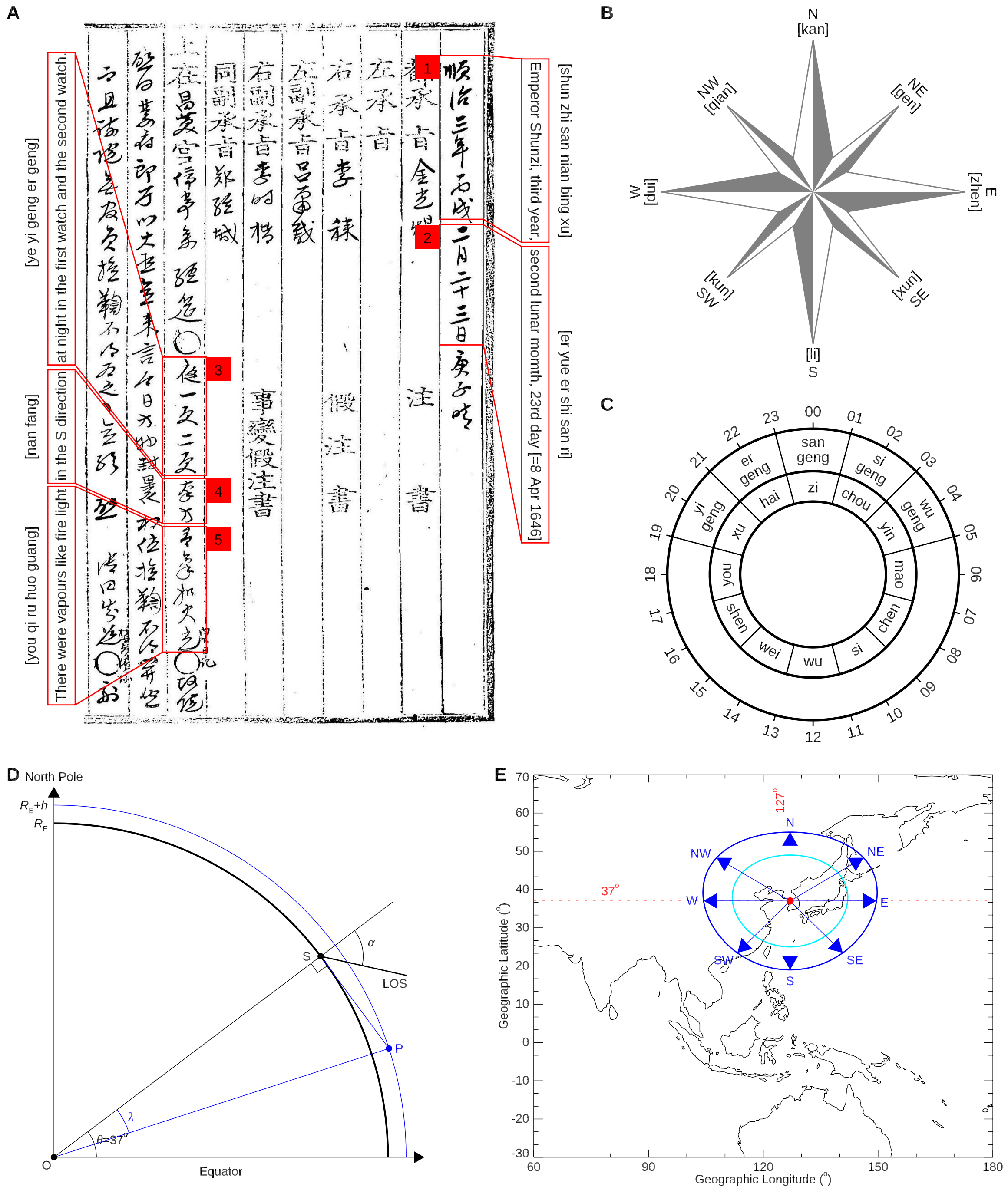

Fig. S3. An example of ancient Korean auroral record and observing geometry. (**A**) Text from the *Sunjongwon Ilgi* illustrating a red aurora observation. (**B**) Definition of local directions. (**C**) Definition of LTs. (**D**) Illustration of the LOS at altitude *h*. The thick black arc and the blue arc represent the Earth's surface and the surface at altitude *h*, respectively. Point O denotes the Earth's center. Point S denotes the location of Seoul at 37° N, 127° E. Point P denotes the maximum visible distance for the surface at altitude *h*. The viewing angle of a LOS relative to local zenith is denoted by α. (**E**) Visible range for altitudes of 350 km (blue) and 150 km (cyan), respectively. The eight arrows define the local directions of red auroral records in Korean historical books (**B**) with 'N', 'NE', 'E', 'SE', 'S', 'SW', 'W', 'NW' for north, northeast, east, southeast, south, southwest, west, northwest, respectively.

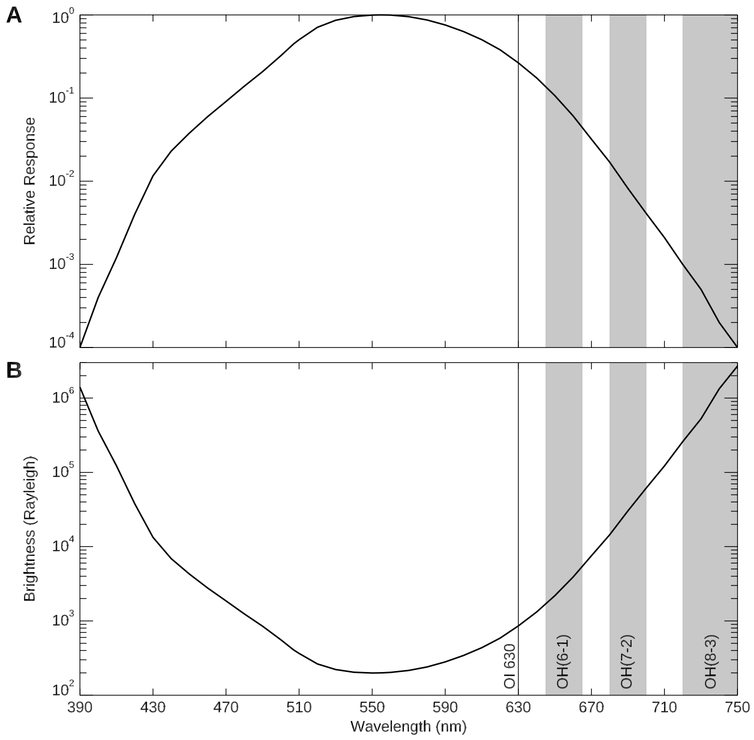

Fig. S4. CIE response function of human eye for photopic vision. (**A**) Relative response function which peaks at 555 nm. (**B**) Threshold of brightness scaled to 200 Rayleigh at 555 nm. Typical emission lines or bands in the red portion of the visible spectrum are also shown in the figure.

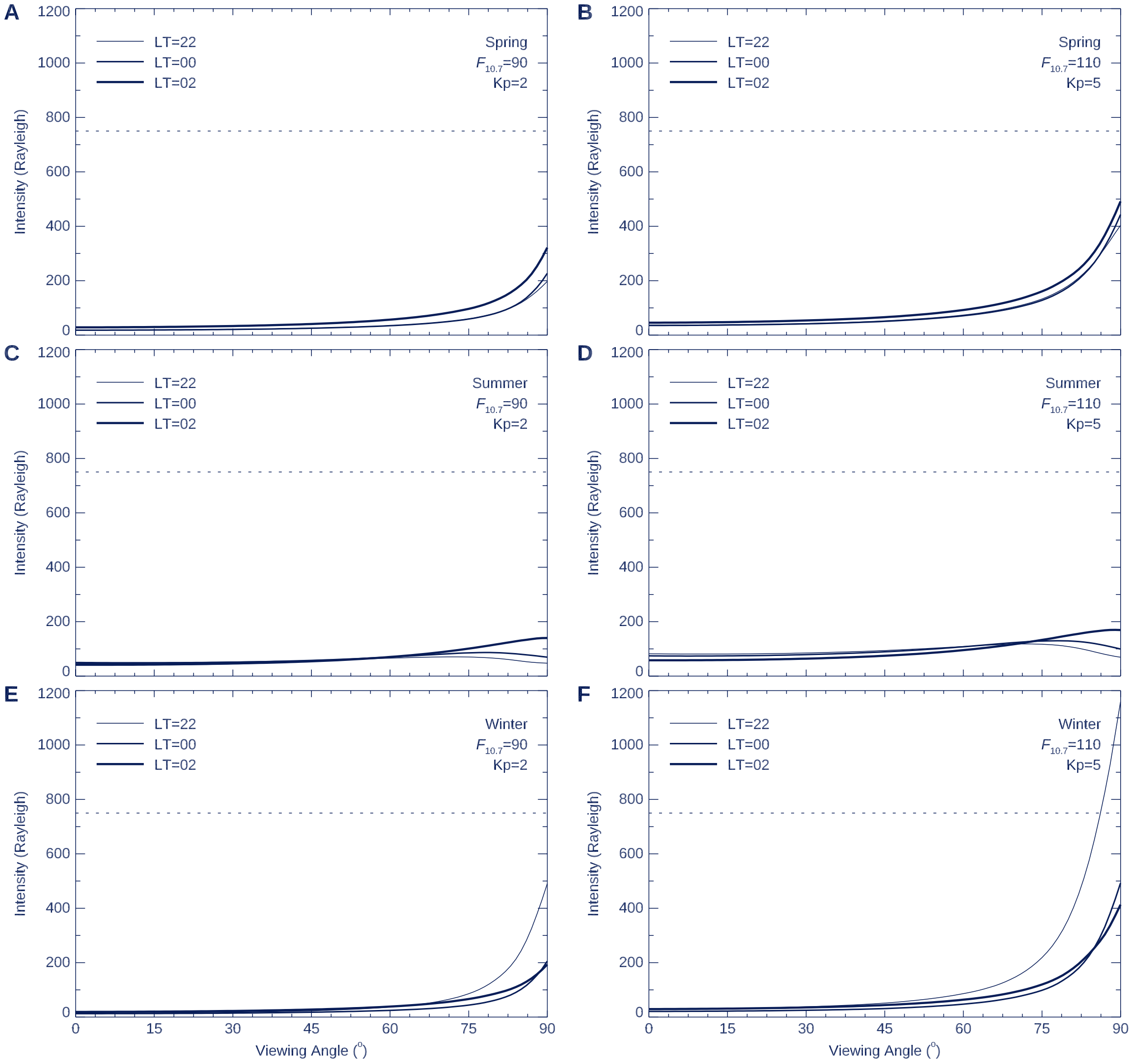

Fig. S5. Intensity variations of redline emission. The left panels are for $F_{10.7}$=90 sfu and Kp=2, and the right panels are for $F_{10.7}$=110 sfu and Kp=5. The solar activity, season, LT, and geomagnetic activity are shown in each panel. The dashed line in each panel indicate a threshold of 750 Rayleigh.

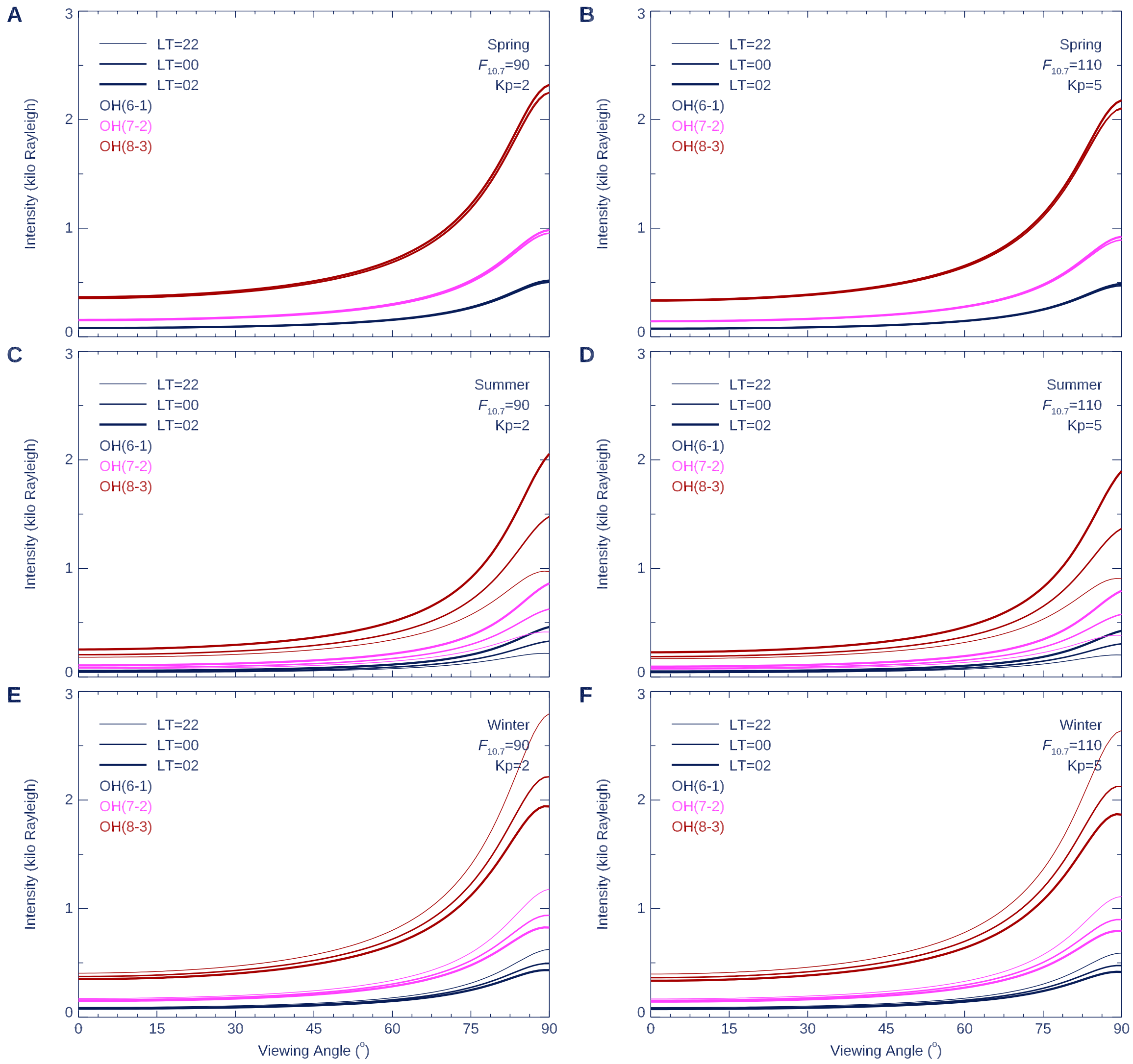

Fig. S6. Intensity variations of OH emission bands. The blue, magenta, and red curves represent the (6→1) band, the (7→2) band, and the (8→3) band, respectively. The solar activity, season, LT, and geomagnetic activity are shown in each panel.

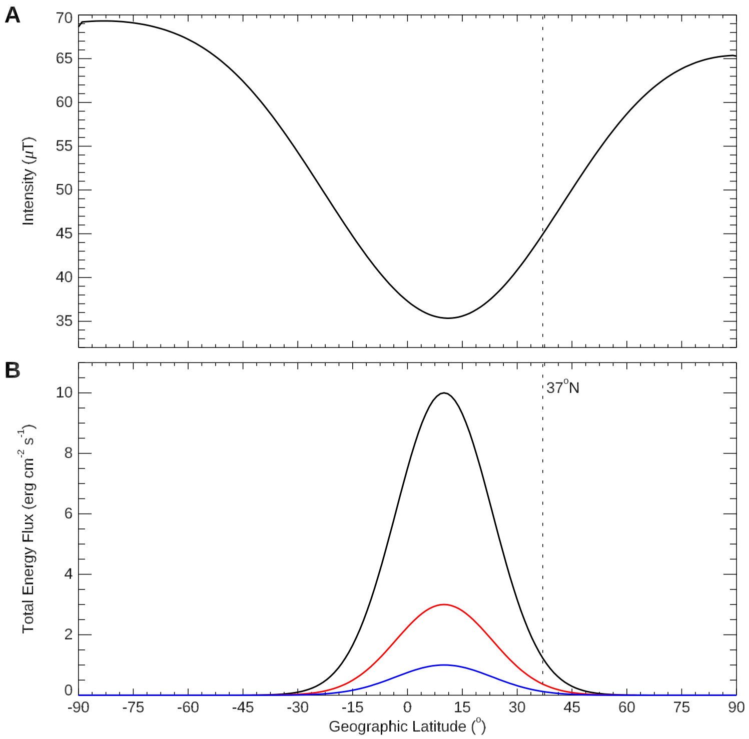

Fig. S7. Variations of magnetic field strength and total input energy flux across the WPA. (**A**) Latitudinal profile of the magnetic field strength at 127° E. (**B**) Gaussian-type latitudinal profile of the total input energy flux at 127° E, with black, red, and blue curves peaking at 10 erg cm^{-2} s^{-1}, 3 erg cm^{-2} s^{-1}, and 1 erg cm^{-2} s^{-1}, respectively.

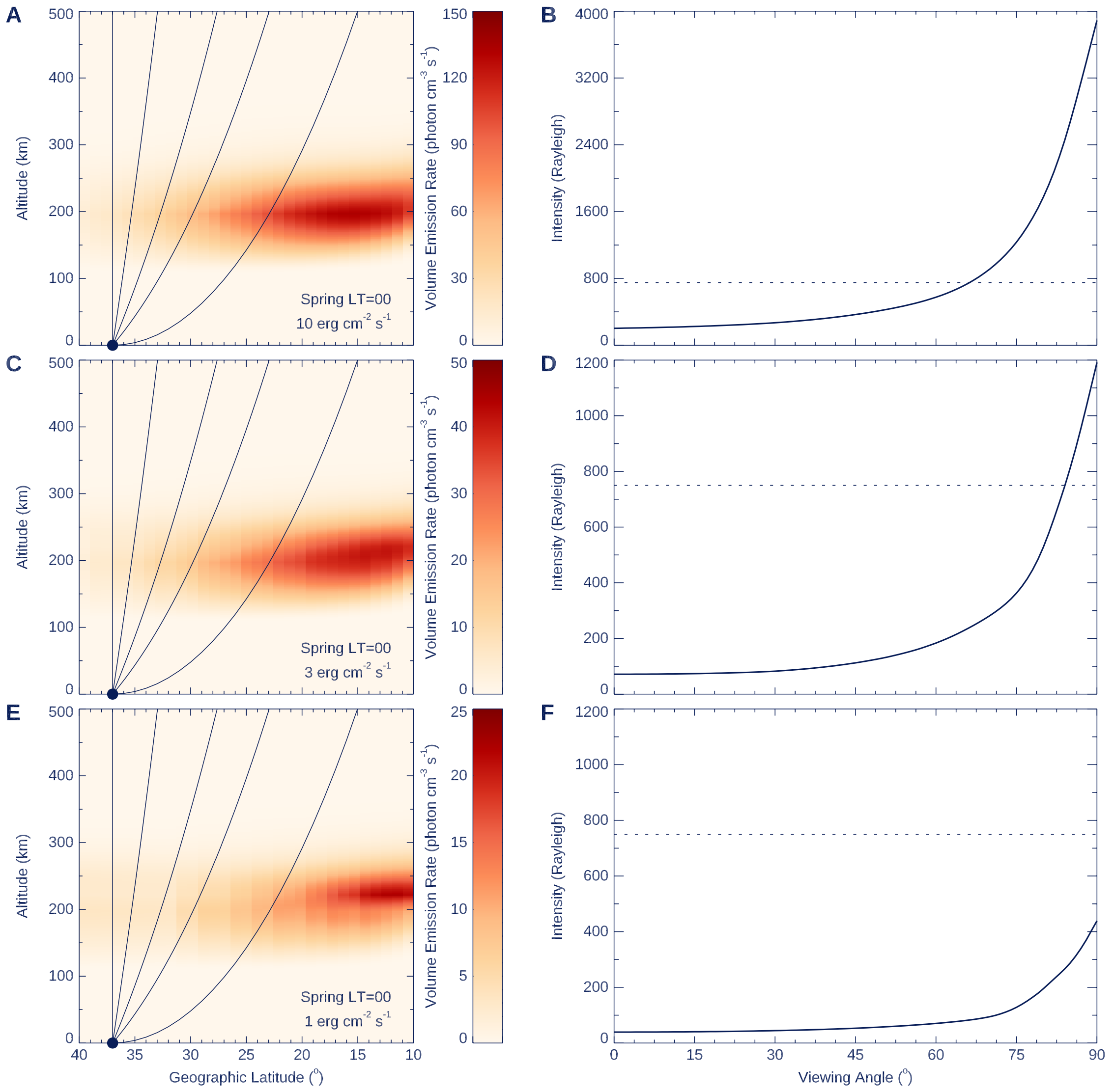

Fig. S8. VERs and emission intensities for different total input energy fluxes. The left panels show the VER for peak energy flux of (**A**) 10 erg cm^{-2} s^{-1}, (**C**) 3 erg cm^{-2} s^{-1} and (**E**) 1 erg cm^{-2} s^{-1}, respectively. The corresponding emission intensities as a function of viewing angle α (defined in *SI Appendix*, Fig. S3*D*) are shown in the right panels (**B**, **D**, and **F**). Other model parameters are all kept at LT=0 h in spring, $F_{10.7}$=70 sfu, and Kp=2.

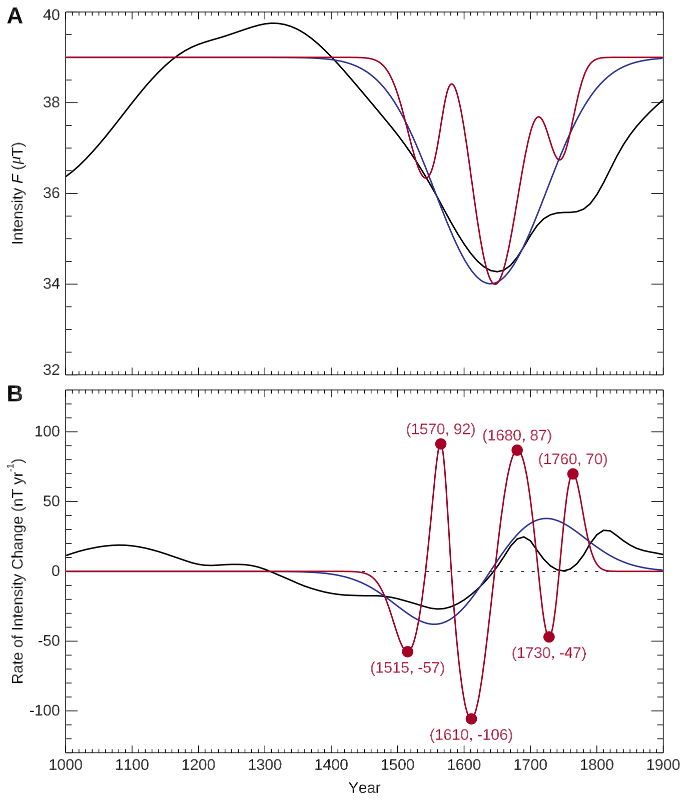

Fig. S9. Predicted evolution of the magnetic field at the WPA center point. (**A**) Predicted magnetic field intensity at the WPA center point (127° E and 10° N). (**B**) Rate of intensity change in (**A**). The blue curve is predicted with the envelope of the auroral records (Fig. 1*D*, magenta dotted curve), and the red curve is predicted with the smoothed auroral frequency (Fig. 1*D*, blue dotted curve). The maxima and minima of the rate of intensity change are marked by red dots in (**B**), and the corresponding years and rates shown in the brackets, respectively. The black curves are derived with the CALS10k.2 model.

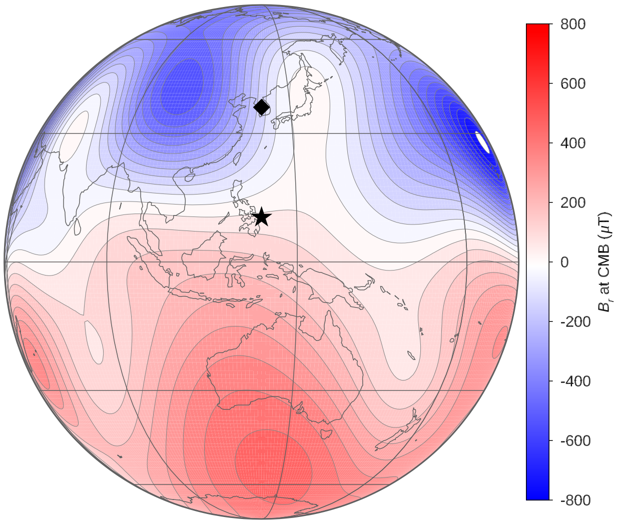

Fig. S10. The radial magnetic field at the CMB given by SHAWQ2k model. The projections of the centre point of the WPA and the location of the auroral observations in Korea onto the CMB are marked as a star and a diamond, respectively.
